# Review of claudin proteins as potential biomarkers for necrotizing enterocolitis

**DOI:** 10.1007/s11845-020-02490-2

**Published:** 2021-01-25

**Authors:** Victoria Griffiths, Niazy Al Assaf, Rizwan Khan

**Affiliations:** 1 Graduate Entry Medical School, University of Limerick, Limerick, Ireland; 2 Department of Neonatology, University Maternity Hospital Limerick, Limerick, Ireland

**Keywords:** Biomarker, Claudin, Intestinal permeability, Necrotizing enterocolitis, Tight junction

## Abstract

**Background:**

Claudin proteins are a component of tight junctions found in cell-cell adhesion complexes. A central feature of necrotizing enterocolitis (NEC) is intestinal permeability, with changes to claudin proteins potentially contributing to intestinal instability, inflammation, and the progression of NEC. A current area of interest is the development of a novel, noninvasive biomarker for the detection of NEC in neonates at risk of developing this disease, in order to reduce morbidity and mortality through earlier intervention.

**Aims:**

This review aims to explore the relevance of claudin proteins in the pathophysiology of NEC and their potential usefulness as a biomarker.

**Methods:**

This review was conducted using the search terms “claudin” + “necrotizing enterocolitis”, with 27 papers selected for review.

**Results:**

Claudin proteins appear to have a role in the stability of the gut epithelium through the regulation of intestinal permeability, maturity, and inflammation. Formula feeding has been shown to promote inflammation and result in changes to claudin proteins, while breastfeeding and certain nutritional supplements lead to reduced inflammation and improved intestinal stability as demonstrated through changes to claudin protein expression. Preliminary studies in human neonates suggest that urinary claudin measurements may be used to predict the development of NEC.

**Conclusions:**

Alterations to claudin proteins may reflect changes seen to intestinal permeability and inflammation in the context of NEC. Further research is necessary to understand the relevance of claudin proteins in the pathophysiology of NEC and their use as a biomarker.

## Introduction

Necrotizing enterocolitis (NEC) is a severe inflammatory bowel disease affecting preterm neonates born with birth weights less than 1500 g, at an incidence of 7–10% and up to 30% mortality [1]. Risk factors for NEC consistently include low birth weight and prematurity, as well as formula feeding and intestinal dysbiosis [2]. Despite being described nearly 40 years ago, a clear understanding of the pathophysiology of NEC is lacking [3]. A reliable and specific biomarker for the prediction or early diagnosis of NEC is an area of current interest, with a recent review highlighting the importance of researching “gut-associated specific biomarkers” for clinical application [4].

The intestinal epithelial surface is the primary barrier protecting the body against invasion of pathogens introduced by the gastrointestinal tract, and is formed by an epithelial layer stabilized by tight junctions [5]. Tight junctions are cell-cell adhesion complexes found on the apical portion of intestinal epithelial cells [6, 7]. Tight junctions are composed of 50 specific proteins, the major two being occludin and claudin proteins which are anchored to the actin cytoskeleton by scaffolding proteins known as zona occludens [8].

This review of literature focuses on the role of claudin proteins in relation to intestinal stability in the context of NEC. The use of claudin proteins as potential biomarkers was considered based on evidence of correlations between claudin protein expression changes seen in human preterm neonates and animal models with clinical features consistent with NEC.

## Methods

Articles were searched using the following databases: Pubmed, Web of Science, and ScienceDirect. Guidelines looked up on included PRISMA, MOOSE, and Cochrane Handbook of Systematic Reviews of Interventions.

Search terms used were “claudin” + “necrotizing enterocolitis.” A search filter was applied for studies performed between 2010 and 2019. All studies in the review were selected using these databases; none was hand-selected. Studies relating to the claudin tight junction and necrotizing enterocolitis were selected. The most recent search was performed on December 06, 2019.

This search yielded 110 results. The most recent work done on tight junction proteins in relation to NEC has been done on mammalian animal models. Therefore, it was decided to concentrate on studies performed on recognized animal models and humans rather than cell-based experimental studies.

Inclusion criteria were as follows: Studies performed between 2010 and 2019 to highlight recent work in this area, studies performed on above terminologies along with overlapping terminologies, and studies focusing on human NEC or mammalian models of NEC.

Exclusion criteria were as follows: Studies performed in 2009 or prior, studies performed in 2020, studies that focused on research methodology, and studies performed using cell/molecular models or non-mammalian species. Excluded studies were determined based upon review of article abstracts or summaries.

Following the application of these criteria, 27 papers were selected for review. The relationship of tight junction changes, specifically claudin proteins, seen in both human and animal models with similar characteristics to human NEC was focused on during this review, and a summary of the results is provided in Table 1. The results are categorized based on appreciated associations with the development of NEC in humans: (1) intestinal prematurity and permeability, (2) gut microbiota and probiotic supplementation, (3) intestinal inflammation and injury, (4) breast milk, and (5) systemic associations seen in extraintestinal tissues. This categorization is to allow for a clearer breakdown of how claudin changes may be involved in the pathogenesis of NEC, and how changes seen with intestinal and extraintestinal systems may be correlated. This could allow the development of a noninvasive biomarker of intestinal disease utilizing claudin changes.

**Table 1:** Summary of claudin isoform changes seen in animal and human models of NEC.

Study	Model	Claudin findings
Prematurity		
Bein et al. (2018)	Human	↓ Claudin-4 gene expression appreciated in human preterm neonates with NEC.
Ferretti et al. (2017)	Human	↓ Claudin-1, ↑ claudin-2, and ↓ claudin-7 seen in human preterm neonatal interstitial explants treated with indomethacin (INDO) was associated with barrier disruption, mediated through the highly expressed nitric oxide synthase (NOS2) enzyme, whereas EGF was shown to have a protective effect through a similar pathway.
Ares et al. (2019)	Rat	↓ Claudin-1, ↑ claudin-2, ↓ claudin-3, and ↓ claudin-4 expression seen in a rat model which was consistent with increased barrier permeability
Bergmann et al. (2013)	MouseHuman	↑ Claudin-2 and internalization of claudin-4 shown to precede intestinal permeability in a time-dependent manner in a mouse model, prior to evidence of histological injury. Human tissue samples showed ↑claudin-2 expression in crypts of colonic epithelium and small intestines in NEC.
Gut microbiota		
Bergmann et al. (2013)	Mouse	Claudin-4 expression levels were preserved after administration of *B. infantis*, which correlated with reduced incidence of NEC in the mouse model and a preservation of intestinal permeability.
Ling et al. (2016)	Rat	↑ Claudin-3 expression normalized after administration of Bifidobacterium and was associated with decreased incidence and severity of NEC by reducing gut permeability in a rat model.
Patel et al. (2012)	Mouse	↑ Claudin-3 expression associated with colonization of commensal bacterial and enteral administration of *Lactobacillus* in the mouse model, suggesting role for claudin-3 in the stability of the intestinal barrier through maturation of tight junctions.
Jakaitis and Denning (2017)	Mouse	↑ Claudin-3 expression in the first 2–3 weeks of life in a mouse model which correlated with intestinal maturity and was potentially upregulated by the presence of both live- and heat-killed LGG through modification of tight junctions.
Intestinal inflammation		
Formula		
Siggers et al. (2011)	Pig	↑ Claudin-1 in response to enteral feeding of formula following 2 days of TPN, in contrast to colostrum feeding which reflected no change from the baseline.
Ravisankar et al. (2018)	Mouse	↑ Claudin-2, ↓ claudin-3, ↓ claudin-4, and ↓ claudin-7 expression in mouse model of NEC induced by formula feeding, cold stress, and hypoxia and is associated with elevated apoptosis (PARP), inflammatory markers (NF-κB and TGF-β), and intestinal permeability.
Roy et al. (2018)	Pig	↓ Claudin-2 expression associated with elevated intestinal permeability and inflammation in a newborn pig model fed fermented formula.
Nutrition		
Xu et al. (2018)	Pig	↑ Claudin-1 expression with supplementation of medium-chain TAG (MCT) in diets of piglets compared to maise oil diet. Suggests a protective role for MCT in improving intestinal barrier digestive function following LPS challenge, mediated through changes in claudin proteins
Xiao, Cao, et al. (2016)	Pig	↑ Claudin-1 expression in the jejunum following supplementation with dietary anemonin with alleviation of LPS-induced intestinal injury and inflammation
Zhu et al. (2018)	Pig	↑ Claudin-1 expression in intestines when supplemented with flaxseed oil, associated with intact epithelial barrier and reduced inflammation in pig model exposed to LPS.
Xiao et al. (2018)	Mouse	↑ Claudin-1 expression and lower inflammatory markers in response to vitamin A treatment in the mouse model. Low vitamin A levels are associated with human NEC.
Xiao, Jiao, et al. (2016)	Pig	Prevention of LPS-induced claudin-1 downregulation following administration of whey protein concentrate (WPC) and inflammation in jejunal mucosa. Suggests that WPC may help attenuate LPS-induced intestinal damage and stabilize the mucosal barrier.
Li et al. (2013)	Pig	↑ Claudin-4 expression in a pig model fed conventional formula, correlated with intestinal permeability and increases in response to epithelial damage in the preterm pig model. Normalization of claudin-4 expression with supplementation of whey protein concentrate
Enzymes and peptides		
Seo et al. (2019)	Mouse	↓ Claudin-3 gene expression in NEC mouse model, with administration of vasoactive intestinal peptide (VIP) leading to ↑ claudin-3 gene expression in the crypts of NEC animals.
Rentea et al. (2012)	Rat	↓ Claudin-1 and ↑ claudin-3 expression correlated with decreased intestinal permeability and severity of NEC with supplementation of intestinal alkaline phosphatase in the rat model
Ares et al. (2019)	Rat	Rho kinase inhibition may have a protective role by normalizing claudin-2 expression in a rat model.
Ischemia		
Jensen et al. (2017)	Mouse	Preservation of claudin-1 levels and intestinal stability in a mouse model given human adipose–derived stromal stem cell (hASC) therapy, likely due to improved mesenteric perfusion and intestinal mucosal integrity.
Hogberg et al. (2013)	Rat	↓ Claudin-1, -14, and -15, and ↑ claudin-8 following exposure to hypoxic-reoxygenation therapy to mimic early pathogenesis of NEC.
Breast milk		
Gunasekaran et al. (2019)	Mouse	↑ Claudin-2, ↑ claudin-3, and ↑ claudin-4 ileal expression associated with increased survival and lower intestinal injury in NEC+HA35- and HA35-treated mouse models compared to untreated NEC mouse model. The effect of hyaluronan (HA), a human milk glycosaminoglycan, was shown to reduce intestinal permeability, bacterial translocation, and pro-inflammatory cytokine release.
Shen et al. (2019)	Rat	↑ Claudin-3 expression in the ileum of rat model. Supplementation with lactadherin associated with reduced claudin-3, histological damage, intestinal permeability, and incidence of NEC
Systemic associations		
Garg et al. (2015)	Mouse	↑claudin-1, ↑claudin-2, ↑claudin-3 and ↑claudin-4, ↑claudin-7, and ↑claudin-8 has been appreciated in tight junctions of site-specific regions of the nephron in mouse model induced by formula feeding, hypoxia and cold stress.
Blackwood et al. (2015)	Human	↓ Claudin-2 expression in intestinal epithelium and ↑ claudin-2 expression in urine found to correlate with severity of NEC independent of other conditions in human neonates.
Thuijls et al. (2010)	Human	↑ Claudin-3 in urine of human neonates with suspected NEC that later on developed NEC.

## Results

### Intestinal prematurity and permeability

Understanding how prematurity contributes to the development of NEC and how these changes are reflected through tight junctions may enhance our understanding of the pathogenesis of this disease. Increased gut permeability is a significant feature of NEC, which contributes to the translocation of pathogenic bacteria and intestinal injury. Correlations between claudin protein expression changes and intestinal permeability have been observed. Elevated claudin-2 and reduced claudin-1, -3, and -4 expression was appreciated in a rat model of NEC, with increased barrier permeability seen in accordance with these claudin expression changes [9]. In a mouse model of NEC, an elevation in claudin-2 expression and internalization of claudin-4 was shown to precede intestinal permeability in a time-dependent manner [10]. Elevated claudin-2 expression was shown in the crypts of colonic epithelium and small intestines from surgical samples of human neonatal NEC patients [10]. Reduced claudin-4 gene expression has been appreciated in human preterm neonates with NEC [11]. This change seen in claudin expression in addition to other tight junction genes (ZO-1, occludin, cingulin) suggests that alterations to tight junction proteins are relevant in NEC [11]. Nitric oxide synthase (NOS2) was found to be highly expressed in preterm infants with NEC, and may have a role in mediating epithelial injury [12]. EGF has been shown to have a protective effect on human neonatal intestinal explants leading to improved tight junction integrity, while administration of indomethacin (INDO) was associated with barrier disruption and was associated with reduced claudin-1 and -7 expression, and overexpression of claudin-2 [12].

### Gut microbiota and probiotic supplementation

The presence of commensal bacterial species or administration of probiotics may have an impact on the expression of claudin proteins and the stability of the intestinal barrier. Administration of the probiotic *Bifidobacterium infantis* was shown to attenuate tight junction expression changes by internalizing claudin-4 and subsequently decreasing incidence of NEC in a mouse model [10]. A study revealed that the administration of Bifidobacterium led to reduced claudin-3 expression and reduced intestinal inflammation in a rat model [13].

The expression of claudin-3 in response to *Lactobacillus* has been focused on in mouse models, with elevated expression of claudin-3 being associated with the colonization of commensal bacteria and gut maturation [14, 15]. Claudin-3 expression appears to be upregulated during the first 2–3 weeks of life while the intestine matures, with both live- and heat-killed *Lactobacillus rhamnosus GG* (LGG), potentially accelerating this process of maturation through tight junction modification [14]. Mice with deficient colonization of commensal bacteria had impaired barrier function and reduced claudin-3 expression, which was remedied following administration of both live- and heat-killed LGG with a lower mortality rate seen in the latter [15].

### Intestinal inflammation and injury

#### Role of formula feeding on intestinal inflammation

Formula feeding may be associated with changes to claudin protein expression and correlate with intestinal damage. In response to enteral feeding of formula following 2 days of TPN, elevated claudin-1 transcription was appreciated in a pig model, in contrast to colostrum feeding which reflected no change from baseline [16]. In a mouse model fed formula, elevated claudin-2 and reduced claudin-3, -4, and -7 expression was associated with elevated apoptosis mediated by poly ADP-ribose polymerase (PARP), inflammatory markers (NF-κB and TGF-β) and intestinal permeability [17]. Elevated intestinal permeability and inflammation were appreciated in a newborn pig model induced by feeding fermented formula, which correlated with reduced claudin-2 expression [18].

#### Role of nutritional supplementation on intestinal inflammation

The influence of enteral supplementation of nutritional substances has been explored, with a major focus on changes seen to claudin-1 levels. In pig models, supplementation with medium-chain tri-acyl glycerides (MCT) and dietary anemonin was both shown to improve intestinal inflammation following lipopolysaccharide (LPS)-induced injury and correlated with elevated claudin-1 expression [19, 20]. Flaxseed oil supplementation led to elevated claudin-1 expression in the intestines of a pig model exposed to LPS and correlated with an intact epithelial barrier with reduced inflammation [21]. Low vitamin A (VA) levels have been associated with human NEC. Elevated claudin-1 expression and reduced inflammatory markers have been appreciated in a mouse model of NEC supplemented with VA [22]. Whey protein concentrate (WPC) was also shown to prevent an LPS-induced decrease in claudin-1 expression and inflammation in the jejunal mucosa of a pig model, suggesting a role for WPC in attenuating LPS-induced intestinal damage and stabilization of the mucosal barrier [23]. Additionally, a preterm pig model fed conventional formula correlated with elevated claudin-4 expression, intestinal permeability, and increased epithelial damage, which normalized after supplementation with whey protein concentrate [24].

#### Role of intestinal enzymes and peptide supplementation on intestinal inflammation

The presence of certain enzymes and peptide products may have a role in adjusting the intestinal barrier and influencing the progression of NEC. Reduced claudin-3 gene expression was appreciated in a NEC mouse model, with the administration of VIP subsequently leading to increased claudin-3 gene expression, reduced inflammation, and reduced tight junction disruption in the intestinal epithelium [25]. Reduced claudin-1 and elevated claudin-3 expression levels correlated with reduced intestinal permeability and severity of NEC in a rat model after supplementation with intestinal alkaline phosphatase (IAP) [26]. Inhibition of Rho kinase (ROCK), which affects cell permeability through the regulation of tight junctions, was shown to have a protective role by normalizing claudin-2 expression in a rat model [9].

#### Ischemia and necrosis

The progression of ischemia and necrosis within the intestines of NEC patients may be associated with changes to claudin proteins. Treatment with human adipose–derived stromal stem cell (hASC) therapy in a mouse model resulted in reduced mortality possibly due to improved mucosal integrity and perfusion, which correlated with elevated claudin-1 [27]. Hypoxic-reoxygenation therapy mimicked early pathogenesis of NEC in a rat model, and was associated with the downregulation of claudin-1, -14, and -15 and upregulation of claudin-8 [28].

### Breast milk

Components of breast milk may have a role in physiologically stabilizing the intestinal barrier through changes in the expression of claudin proteins. Elevated claudin-2, -3, and -4 expression in the ileum of NEC mouse models treated with hyaluronan (HA)-35 was associated with increased survival and lower intestinal injury [29]. Supplementation with lactadherin, an immune-related glycoprotein in human milk, leads to reduced claudin-3 expression, intestinal permeability, and incidence of NEC in a rat model [30].

### Systemic associations

Changes seen in claudin proteins have been studied in extraintestinal tissues and correlated with the clinical progression of NEC in humans and animal models. Elevated claudin-1, -2, -3, -4, and -8 has been appreciated in tight junctions of site-specific regions of the nephron in a mouse model induced by formula feeding, hypoxia, and cold stress [31]. Reduced claudin-2 expression in the intestinal epithelium and an elevation in claudin-2 protein in the urine were shown to correlate with the severity of NEC in a small study of human neonates, and was shown to be independent of other conditions including sepsis, medication use, or ventilation [32]. An elevated level of urinary claudin-3, urinary intestinal fatty-acid binding protein (I-FABP), and fecal calprotectin has been appreciated in samples from human neonates with suspected NEC who later went on to develop the disease, with urinary I-FABP being useful in predicting the severity of NEC [33].

## Discussion

Understanding the role that claudins may have on the stability and permeability of the premature gut barrier, and how expression of claudin proteins change in response to a variety of risk factors associated with the development of NEC is the focus of this literature review. By having a clearer idea of claudin proteins in the progression of NEC, this knowledge may be applied to the development of a novel biomarker for this disease in order to predict the severity of NEC, or as a therapeutic target.

Intestinal permeability is a natural phenomenon seen in premature infants to improve intestinal absorption of nutrients from breast milk, including immunoglobulins. Alterations to claudin-1, -2, -3, -4, and -7 have been shown in human and animal models of NEC, and appear to have a central role in contributing to intestinal stability and permeability of the intestinal epithelium [9–12]. Claudin-2 expression changes have been shown to precede changes in intestinal permeability in a mouse model, with elevated claduin-2 expression demonstrated in surgical samples from human NEC [10]. This temporal pattern may encourage future studies to focus on changes in claudin proteins in advance of the development of NEC and may be useful as a biomarker for intestinal injury in infants at risk of developing NEC.

Interactions between the gut microbiota and tight junction stability reflect the dynamic nature of the preterm intestinal barrier. Bacterial dysbiosis has been appreciated in the pathogenesis of NEC, which refers to the imbalance of commensal bacterial and a low diversity of bacteria, leading to the proliferation of pathogenic species contributing to intestinal inflammation [34]. A diverse gut microbiota with the presence of commensal species may have a role in reducing intestinal inflammation and improving intestinal stability through maturation of claudin proteins, particularly claudin-3 and claudin-4 [10, 13–15]. Further research into how commensal species stabilize the intestine through tight junctions may be useful when considering probiotics as a potential therapy in the prevention or treatment of NEC by promoting neonatal intestinal maturity. Additionally, it appears claudins may have a role in stabilizing the intestinal barrier during the first few weeks of life, which may be a useful characteristic while searching for a biomarker that detects the early progression of NEC.

Intestinal inflammation and permeability lead to the translocation of bacteria and a detrimental systemic inflammatory response in premature neonates with NEC. Formula feeding may contribute to intestinal inflammation through modification of the epithelial tight junction barrier. Based on the reviewed studies, formula feeding was shown to result in changes in the expression of claudin-1, -2, -3, -4, and -7 [16–18]. Although there appears to be species-specific differences in whether certain claudins are up- or downregulated, formula feeding was associated with changes to claudin expression, increased inflammation, and intestinal permeability in mouse and pig models of NEC [16–18]. Supplementation with nutritional substrates including MCT, dietary anemonin, flaxseed oil, and WPC was shown to improve intestinal inflammation and intestinal stability in pig models of NEC, with changes primarily seen with claudin-1 expression [19–21, 23]. Additionally, vitamin A which has been observed as being deficient in NEC patients was shown to improve intestinal inflammation in a mouse model, which correlated with elevated claudin-1 [22]. Further research focusing on how the supplementation of certain factors influences the intestinal barrier to improve stability and reduce inflammation may be a potential therapeutic intervention, and may continue to support a role for claudin proteins in mediating these protective effects.

The supplementation of enzymes, including VIP and IAP, and the inhibition of ROCK were shown to have protective effects on the intestinal epithelium by reducing intestinal inflammation and improving tight junction stability through claudin expression changes [9, 25, 26]. Based on these studies, various supplements may have a therapeutic role in adjusting the progression of NEC by modifying tight junction proteins to reduce intestinal permeability and inflammation. Further research on the mechanism by which supplements improve the intestinal barrier through the regulation of claudins supports their use as a marker of intestinal integrity.

Alterations to musical integrity and perfusion may have a role in the progression of NEC. Changes seen in claudin-1, -8, -14, and -15 in response to a hypoxic environment indicate that claudins may be a useful marker for detecting the progression of ischemic damage in NEC patients [27, 28]. Additional studies will be required to understand the role hypoxic damage in the context of NEC, and how claudins may be a useful marker of intestinal damage due to hypoxic injury.

Breastfeeding has appreciated benefits in the prevention of NEC; however, the exact mechanism has yet to be clarified. The role of tight junctions in mediating the beneficial effects of breastfeeding on intestinal stability may be significant. Claudin-2, -3, and -4 appear to be influenced by components of breast milk including hyaluronan and lactacdherin to alter intestinal stability and injury in both mouse and rat models [29, 30]. The direct effect of breast milk on intestinal permeability and protection from the development of NEC may be an area for future studies to explore whether the protective effects of breast milk can be attributed to the regulation of tight junction proteins.

This review highlights the involvement of the kidneys in the multisystem pathophysiology of NEC. Elevated claudin-1, -2, -3, -4, and -8 has been appreciated within tight junctions of the nephron in a mouse model in the context of various risk factors for NEC including formula feeding, hypoxia, and hypothermia [31]. Recent studies using urinary samples to measure claudin proteins in the context of human NEC have promising results. Reduced intestinal claudin-2 and elevated urinary claudin-2 levels were shown to correlate with the severity of NEC in human neonates [32]. Elevated urinary claudin-3, urinary I-FABP, and fecal calprotectin have also been appreciated in human samples that developed NEC [33]. These studies demonstrate a possible link between systemic and intestinal claudin changes, which may be useful in larger studies focusing on noninvasive measurements of intestinal damage which correlates to the clinical progression of NEC. Based on the papers reviewed in this study, changes seen in claudin proteins appear across relevant risk factors for NEC and potentially in a time-dependent manner alongside intestinal permeability, which supports claudin proteins as a promising avenue for future studies towards a biomarker for NEC.

## Conclusion

An appropriate noninvasive clinical biomarker to detect NEC is an area of interest, with claudin proteins being a candidate for future studies. Studies with animal models and human NEC demonstrate a possible correlation between claudin protein expression changes and factors that influence the intestinal barrier, including various understood risk factors for NEC such as intestinal permeability, altered gut microbiota, intestinal inflammation, and breastfeeding. Although these reviewed studies focused primarily on animal models and small studies in human preterm neonates, recent studies support that claudin protein expression changes may be measurable in a noninvasive manner, with correlations between intestinal claudin and urinary claudin changes being a possible biomarker to detect NEC in at risk neonates.

Future studies are required to understand how claudin isoform expression is influenced in the progression of NEC and how this knowledge can be translated to the use of claudins as a biomarker. Future directions include exploring the relationship between disrupted tight junctions and how this directly relates with the inflammation and severity of NEC. Understanding how changes seen in the expression of claudin isoforms changes under a variety of physiological circumstances will contribute towards their use as a biomarker may provide an avenue for the prevention or management of NEC by modulating the immature gut barrier through tight junction modification.

Claudins and other tight junction proteins appear to have a measurable role in the pathogenesis of NEC as a marker of intestinal permeability. Changes in claudin proteins in tissue samples may be a useful clinical marker to detect NEC in early stages to ensure adequate intervention or prevention of NEC in premature neonates at risk for the development of this disease.

## Data Availability

Not applicable.
